# Detection and Zoonotic Implications of Shiga Toxin–Producing *Escherichia coli* in Black Bengal Goats in Bangladesh

**DOI:** 10.1002/vms3.71076

**Published:** 2026-07-10

**Authors:** Shubho Sutradhar, Shormin Akter, Sayeda Ayesha Sultana, Sudeb Sarker, Md. Rigan Molla, Moshammat Naifa Begum, Marina Ghosh, Sharifuzzaman

**Affiliations:** ^1^ Department of Livestock Services Dhaka Bangladesh; ^2^ Department of Microbiology and Veterinary Public Health Chattogram Veterinary and Animal Sciences University Chittagong Bangladesh; ^3^ BRAC Mymensingh Bangladesh; ^4^ Department of Pathology and Parasitology Faculty of Veterinary Medicine Jashore University of Science and Technology Jashore Bangladesh

**Keywords:** Black Bengal goat, *E. coli*, *eae*, *ehxA*, *hlyA*, *saa*, Shiga toxin, *stx1*, *stx2*, *subA*

## Abstract

**Background:**

Shiga toxin–producing *Escherichia coli* (STEC) is an important zoonotic pathogen and a global public health concern due to its association with foodborne disease.

**Objectives:**

This study aimed to determine the prevalence of STEC and general *E. coli* carriage in Black Bengal goats in Bangladesh and assess associated risk factors for zoonotic transmission.

**Methods:**

A total of 300 goats (200 diarrheic and 100 healthy) were sampled from the Shahedul Alam Quadary Teaching Veterinary Hospital and its surrounding areas between July 2023 and June 2024. Recto‐anal junction swabs were cultured on MacConkey agar, Eosin Methylene Blue agar, and cefixime‐tellurite–supplemented Sorbitol MacConkey agar. Sorbitol non‐fermenting colonies were screened for uniplex PCR for *stx1*, *stx2*, *hlyA* and multiplex PCR for *eae*, *ehxA*, *saa*, and *subA* genes using multiplex PCR.

**Results:**

Overall, 4.3% of isolates were sorbitol non‐fermenters. Molecular characterization revealed that one isolate (0.3%) from a diarrheic goat carried the *stx2* and *ehxA* genes, while another isolate (0.3%) from a healthy goat was positive for *saa* and *subA*. No isolates carried *stx1*, *hlyA*, or *eae*. The isolation rate of *E. coli* (non‐STEC) was significantly higher in diarrheic goats than in healthy ones (*p* = 0.001). Risk factor analysis revealed significant associations between general *E. coli* carriage and several factors, including frequent child–animal contact (OR = 2.45, *p* = 0.031), shared living spaces (OR = 2.36, *p* = 0.045), and farms near waterlogged areas (OR = 3.72, *p* = 0.008). Mixed livestock rearing and seasonal humidity also contributed to increased *E. coli* carriage.

**Conclusions:**

These findings, although representing a low overall prevalence, demonstrate the presence of diverse virulence profiles in Black Bengal goats, including markers associated with severe human disease in LEE‐negative strains. The detection of *saa* and *subA* suggests that these goats may serve as a reservoir for atypical STEC strains with zoonotic potential, necessitating broader surveillance strategies.

## Graphical abstract

Low prevalence of STEC was detected in Black Bengal goats in Bangladesh; however, isolates carrying *stx2*, *ehxA*, *saa*, and *subA* highlight the presence of atypical strains with zoonotic potential. Identified risk factors emphasize the need for enhanced surveillance and improved farm hygiene practices to mitigate public health risks.

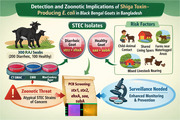

## 1 Introduction

*Escherichia coli* (is a Gram‐negative bacillus commonly found in the intestinal tract of humans and animals. While most strains are harmless commensals, certain pathotypes possess virulence factors that enable them to cause serious disease. Among these, Shiga toxin–producing *E. coli* (STEC) also known as verocytotoxin‐producing *E. coli* is a globally recognized zoonotic pathogen. STEC is responsible for foodborne outbreaks and sporadic cases of haemorrhagic colitis and haemolytic uremic syndrome (HUS), conditions associated with high morbidity and mortality (P. M. Griffin and Tauxe 1991; Brett et al. 2003). The primary virulence factor, Shiga toxin, closely resembles the cytotoxin produced by *Shigella dysenteriae* (Staats et al. 2003), and both O157:H7 and non‐O157 serogroups have been implicated in human disease (Orden et al. 2008).

Domestic ruminants, particularly cattle, are considered the principal reservoirs of STEC. However, other ruminants such as goats, sheep, and deer have also been shown to harbour these pathogens, contributing to environmental contamination and zoonotic transmission (Caprioli et al. 2005; Fegan and Desmarchelier 1999). Human infection can occur through direct contact with animals, consumption of contaminated food or water, or exposure to faecal matter (Erickson and Doyle 2007). In smallholder farming systems, where close human–animal interactions are common, the risk of zoonotic transmission is significantly elevated.

In Bangladesh, goats play a vital role in rural livelihoods, contributing to income generation, nutrition, and food security. The country is home to over 34.5 million goats, with Black Bengal goats comprising approximately 90% of the population (Rischkowsky and Pilling 2007). These animals are predominantly reared under smallholder systems, often in close proximity to humans, particularly women and children raising concerns about zoonotic disease transmission. Despite the documented prevalence of STEC in cattle within Bangladesh (Islam 2012; Islam et al. 2008), data on STEC carriage in goats, especially the Black Bengal breed, remain scarce. Furthermore, understanding the prevalence and risk factors associated with general *E. coli* carriage in these animals is crucial, as *E. coli* serves as a key indicator of faecal contamination and potential exposure to a range of enteric pathogens, including STEC, in such close‐contact environments.

Given the public health implications and the lack of surveillance data, this study was undertaken to assess the prevalence of STEC and associated zoonotic risk factors for both STEC and general *E. coli* carriage in both healthy and clinically ill Black Bengal goats. The findings aim to inform future disease control strategies and contribute to the broader understanding of STEC epidemiology and general *E. coli* dissemination in mixed farming systems.

## 2 Materials and Methods

### 2.1 Study Area and Collection of Faecal Samples

A cross‐sectional survey was conducted between July 2023 and June 2024 to isolate potential STEC from Black Bengal goats. The study was carried out at the Shahedul Alam Quadary Teaching Veterinary Hospital (SAQTVH), Chattogram Veterinary and Animal Sciences University (CVASU), and surrounding rural areas in Chattogram, Bangladesh.

A total of 300 goats were sampled, comprising 200 clinically sick (diarrheic) and 100 apparently healthy individuals. Goats were classified as clinically sick based on the presence of watery faeces, a moist anal region, and elevated rectal temperature (>102.5°F). Faecal samples were collected by inserting a sterile cotton swab into the recto‐anal junction (RAJ) and gently swabbing the mucosal wall to obtain faecal material.

Each swab was immediately placed into a sterile 5‐mL tube containing buffered peptone water (Oxoid, Basingstoke, Hampshire, UK) and transported under chilled conditions to the Poultry Research and Training Centre at CVASU for microbiological analysis.

### 2.2 Data Collection

Farms were selected using a stratified random sampling approach to ensure representation across diverse management systems, environmental exposures, and human–animal interaction patterns. In addition to sample collection, demographic and epidemiological data were recorded for each goat using a structured data collection sheet. Information was obtained through direct interviews with animal owners, covering variables such as age, sex, health status, feeding practices, water source, antibiotic and antiparasitic use, manure management, and proximity to environmental risk factors.

Data on the use of antibiotics and antiparasitic drugs were collected by recording whether these medications were used based on a veterinary prescription or without a prescription. Information on manure management practices was collected by recording the method of manure disposal, which was categorized as either open disposal or composting. Human–animal interaction was evaluated through household surveys and observational checklists. Key indicators included the frequency of contact between goats and children or women. The contact frequency was categorized as high (≥3 direct interactions per day or ≥15 per week) or low (≤2 interactions per day or <15 per week). Behavioural logs and photographs were used to support observations. Environmental data were collected to assess proximity to waterlogged areas or wetlands and no wetlands. The presence of other livestock species was recorded through direct observation and owner reports, noting species diversity such as cattle, poultry, and sheep. Vector (fly) density was estimated by placing sticky traps near animal housing to count flies, and by physically examining goats and surrounding areas. Vector density was categorized as low (<20 flies/trap/day) or high (≥20 flies/trap/day) based on counts per unit area.

Seasonal data including temperature, humidity, and rainfall were obtained from the Bangladesh Meteorological Department (BMD 2024), and seasonal classifications (e.g., monsoon, dry season) were used to correlate environmental conditions with vector density (fly) and *E. coli* prevalence.

### 2.3 Bacteriological Investigation

Faecal swab samples collected in buffered peptone water (Oxoid, Basingstoke, Hampshire, UK) were incubated at 37°C for 24 h to allow enrichment, following standard pre‐enrichment protocols for enteric pathogens (ISO 16654:2001). After incubation, each sample was streaked onto MacConkey agar and incubated at 37°C for an additional 24 h. Colonies exhibiting large pink morphology were considered lactose fermenters (Cheesbrough 2006). Representative colonies were subsequently subcultured onto Eosin Methylene Blue (EMB) agar (Oxoid) and incubated under identical conditions. Colonies producing a characteristic metallic green sheen on EMB agar were presumptively identified as *E. coli* (MacFaddin 2000).

Selected *E. coli* colonies were further streaked onto 5% bovine blood agar to ensure purity, following standard microbiological protocols (Cheesbrough 2006). From these purified cultures, at least five colonies per sample were inoculated into Brain Heart Infusion (BHI) broth and incubated at 37°C for 24 h to promote optimal bacterial growth (MacFaddin 2000). For long‐term preservation and downstream molecular analysis, cultures were stored at −80°C in BHI broth supplemented with 15% glycerol, a widely accepted cryoprotectant for bacterial stocks (Sambrook and Russell 2001).

To assess sorbitol fermentation, preserved *E. coli* isolates were first revived on 5% bovine blood agar to ensure viability and purity (Cheesbrough 2006). Growth from these plates was streaked onto cefixime‐tellurite sorbitol MacConkey (CT‐SMAC) agar, prepared according to manufacturer instructions. Briefly, 25.75 g of sorbitol MacConkey agar was dissolved in 500 mL of distilled water, autoclaved, and cooled to 50°C in a water bath. Cefixime (0.05 mg/mL) and potassium tellurite (1.25 mg/mL) were added aseptically, and 20 mL of the medium was dispensed into sterile Petri dishes. CT‐SMAC is widely used for selective isolation of *E. coli* O157:H7 due to its inability to ferment sorbitol, resulting in colourless colonies (Bennett et al. 1995).

CT‐SMAC plates were incubated at 37°C for 18–24 h. Sorbitol non‐fermenting colonies appeared colourless and were phenotypically suspected as potential STEC isolates (Wells et al. 1983; March and Ratnam 1986; Krishnan et al. 1987).

### 2.4 Molecular Identification of Shiga Toxin–Producing *E. coli*

#### 2.4.1 Detection of Virulence Genes

Sorbitol non‐fermenting *E. coli* isolates were screened for the presence of Shiga toxin genes (*stx1* and *stx2*), the haemolysin gene (*hlyA*), intimin (*eae*), enterohemolysin (*ehxA*), STEC autoagglutinating adhesin (*saa*), and subtilase cytotoxin (*subA*) using multiplex polymerase chain reaction (PCR). Primer sequences, annealing temperatures, and expected amplicon sizes are listed in Table 1.

**TABLE 1:** Oligonucleotide primers used for detection of *stx1*, *stx2*, and *hlyA* genes.

Primer	Primer sequence (5′–3′)	Target gene	Size of product (bp)	Reference
*hly* F *hly* R	ACG ATG TGG TTT ATT CTG GA CTT CAC GTG ACC ATA CAT AT	*hly*	165	Ogawa et al. (2001)
*stx*1F *stx*1 R	ACA CTG GAT GAT CTC AGT GG CTG AAT CCC CCT CCA TTA TG	*stx*1	614	
*stx*2 F *stx*2 R	CCA TGA CAA CGG ACA GCA GTT CCT GTC AAC TGA GCA GCA CTT T	*stx*2	779	
*eae* F *eae* R	F: GACCCGGCACAAGCATAAGC R: CCACCTGCAGCAACAAGAGG	*eae*	384	Paton and Paton (1998)
*ehxA* F *ehxA* R	F: GCATCATCAAGCGTACGTTCC R: AATGAGCCAAGCTGGTTAAGCT	*ehxA*	534	Paton and Paton (1998)
*saa* F *saa R*	F: CGTGATGAACAGGCTATTGC R: ATGGACATGCCTGTGGCAAC	*saa*	119	Paton and Paton (2002)
*subA* F *subA* R	F: TATGGCTTCCCTCATTGCC R: TATAGCTGTTGCTTCTGACG	*subA*	556	Paton et al. (2004)

#### 2.4.2 PCR Amplification and Gel Electrophoresis

DNA templates were prepared by boiling a loopful of fresh colonies in 200 µL of deionized water at 99°C for 15 min, followed by immediate cooling on ice. The suspension was centrifuged at 15,000 rpm for 2 min, and 100 µL of the supernatant was used as template DNA.

For the amplification of *hlyA*, *stx1*, and *stx2* genes, gene‐specific primers and a master mix (Takara, Japan) were used. Each 50 µL PCR reaction contained 250 nM of each primer mix and 1 µL of DNA template. PCR amplification was performed using a thermal cycler (Applied Biosystems 2720, Singapore). Each reaction was subjected to 30 cycles under optimized conditions. For the *hlyA* gene, the thermal profile included an initial denaturation at 95°C for 3 min, followed by 20 s of denaturation at 95°C, 40 s of annealing at 58°C, and 1 min of extension at 72°C. A final extension step was carried out at 72°C for 1 min, followed by a hold at 4°C. For *stx1* and *stx2* genes, the protocol was similar, with the exception of a slightly longer denaturation step of 30 s at 95°C.

The multiplex PCR was performed for amplification of *eae*, *ehxA*, *saa*, and *subA* genes in a 50‐µL reaction mixture containing 250 nM of each primer mix and 1 µL of DNA template. The thermal profile included an initial denaturation at 95°C for 5 min, followed by 35 cycles of 95°C for 45 s, 58°C for 45 s, and 72°C for 1 min, with a final extension at 72°C for 10 min.

Amplified products were resolved by electrophoresis on a 1% agarose gel (Seakem LE agarose, Lonza) prepared in 1× TAE buffer and stained with ethidium bromide at a concentration of 5 µg/mL. Each PCR product (5 µL) was mixed with 1 µL of 6× loading dye and loaded into individual wells. A 1‐kb DNA ladder (Thermo Scientific O'GeneRuler 1 kb Plus) was used as a molecular size marker. Electrophoresis was conducted at 110 volts for 20 min. Gels were rinsed and visualized under ultraviolet illumination using a BDA Digital Transilluminator (Biometra GmbH, Germany). Amplicon sizes of 165 bp (*hlyA*), 614 bp (*stx1*), 779 bp (*stx2*), 534 bp (*ehxA*), 119 bp (*saa*), and 556 bp (*subA*) confirmed the presence of respective genes in the isolates.

### 2.5 Statistical Analysis

All collected data were verified for completeness and accuracy before being entered into a spreadsheet using Microsoft Excel 2007 (Microsoft Corporation, USA). Statistical analyses were performed using STATA version 12 (StataCorp LP, College Station, TX, USA). Descriptive statistics were used to summarize categorical variables, including proportions and 95% confidence intervals (CI). Associations between *E. coli* positivity and independent variables (e.g., age, sex, health status, feeding practices, environmental exposure) were assessed using the chi‐square (χ^2^) test. A *p* value of less than 0.05 was considered statistically significant.

## 3 Results

### 3.1 Isolation Rates and Virulence Profile of *E. coli*

Of the 300 Black Bengal Goats investigated, 169 were found to be positive for *E. coli* after culturing through MacConkey agar. The isolation rate of *E. coli* (non‐STEC) was significantly higher in diarrheic goats (110/200) than in healthy ones (59/100) (*p* = 0.001). Among these *E. coli* positive samples, 117 produced a metallic sheen on EMB agar, and 13 were identified as sorbitol non‐fermenting *E. coli* on CT‐SMAC agar.

**FIGURE 1:**
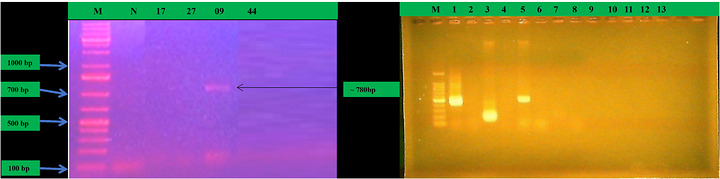
Left panel: Uniplex PCR amplification for *stx1*, *stx2*, and *hlyA* from a sorbitol non‐fermenting *E. coli* isolate. Lane M: 100‐bp DNA ladder (GeneRuler 1 kb Plus, Thermo Scientific); Lane N: negative control; Lane 09: positive isolate. Right panel: Multiplex PCR amplification for *eae*, *ehxA*, *saa*, and *subA* genes. Lanes 1–13 represent individual samples. Lane 1 is positive for *ehxA* (534 bp), Lane 3 for *saa* (119 bp), and Lane 5 for *subA* (556 bp).

Molecular characterization (Figure 1) of the 13 sorbitol non‐fermenting *E. coli* isolates revealed that two (0.67% of total goats) were confirmed to carry at least one virulence marker. One isolate from a clinically sick goat was positive for both *stx*2 and *ehx*A. Another isolate from an apparently healthy goat carried the saa and *subA* genes but was negative for *stx* and *eae*. All 13 isolates were negative for *stx*1, *eae*, and *hly*A (Table 2).

**TABLE 2:** Comparative isolation rates of *E. coli* and sorbitol non‐fermenting *E. coli* in Black Bengal goats (*n* = 300).

Status	MacConkey positive	EMB positive	CT‐SMAC	
			Fermenting	Non‐fermenting
Clinically sick—diarrheic (*n* = 200)	110	92	60	9
Apparently healthy (*n* = 100)	59	25	20	4
Total (*n* = 300)	169	117	80	13

### 3.2 Demographic and Health Variables

Adult goats showed a markedly higher positivity rate (84%) compared to kids (16%), although the difference was not statistically significant (*p* = 1.00). Female goats had a higher isolation rate (62%) than males (38%), but this difference was also not significant (*p* = 0.92). A strong association was observed between health status and *E. coli* carriage: diarrheic goats had a significantly higher positivity rate (79%) compared to healthy animals (21%) (*p* = 0.001).

Body condition score (BCS) was a significant predictor of *E. coli* positivity. Cachectic goats exhibited the highest isolation rate (79%), followed by RBC (14%), CPD (7%), and GBC (1%) (*p* = 0.001), indicating a strong correlation between poor body condition and bacterial shedding (Table 3).

**TABLE 3:** Detection rates of *E. coli* in recto‐anal junction (RAJ) swabs from Black Bengal goats across different variables (*n* = 300).

Variable	Total animal (N)	Proportion (positive)	95% CI	*p* value
Age	Kid (70)	0.16 (19)	0.10–0.24	1.00
	Adult (230)	0.84 (98)	0.75–0.90	
Sex	Male (127)	0.38 (45)	0.3–0.48	0.92
	Female (173)	0.62 (72)	0.52–0.70	
Health status	Diarrheic (200)	0.79 (92)	0.7–0.85	0.001
	Healthy (100)	0.21 (25)	0.14–0.30	
Only stall feeding	Yes (47)	0.08 (9)	0.04–0.14	0.92
	No (253)	0.92 (108)	0.86–0.96	
Green grass provided	Yes (163)	0.56 (65)	0.46–0.64	0.18
	No (137)	0.44 (52)	0.35–0.53	
Supply of water	Pond (192)	0.67 (78)	0.57–0.75	0.13
	Tube well (108)	0.33 (39)	0.25–0.43	
Body score	GBC (18)	0.01 (1)	0.0002–0.05	0.001
	RBC (46)	0.14 (16)	0.08–0.21	
	CPD (32)	0.07 (8)	0.03–0.13	
	Cachectic (204)	0.79 (92)	0.70–0.86	
Antibiotic used	Yes (80)	0.15 (18)	0.09–0.23	0.21
	No (220)	0.85 (99)	0.77–0.91	
Manure management	Open disposal (190)	0.72 (137)	0.65–0.78	0.003
	Composting (110)	0.28 (31)	0.20–0.37	
Antiparasitic use	Prescribed (130)	0.32 (42)	0.24–0.41	0.02
	Non‐prescribed (170)	0.68 (116)	0.60–0.75	
Human–animal contact	High contact (150)	0.66 (99)	0.58–0.74	0.0
	Low contact (150)	0.34 (51)	0.26–0.42	
Environmental exposure	Wetland proximity (120)	0.71 (85)	0.62–0.79	0.01
	No wetland (180)	0.29 (52)	0.22–0.36	
Seasonal data	Monsoon (150)	0.69 (104)	0.61–0.76	0.005
	Dry season (150)	0.31 (46)	0.24–0.39	

### 3.3 Feeding and Water Supply

Goats receiving only stall feeding had a significantly lower positivity rate (8%) compared to those with mixed feeding practices (92%) (*p* = 0.92). Provision of green grass did not show a significant effect on *E. coli* carriage (*p* = 0.18), although goats receiving green grass had a slightly higher positivity rate (56%) than those without (44%). Water source appeared to influence bacterial prevalence, with goats drinking from ponds showing a higher positivity rate (67%) than those supplied by tube well (33%), though the difference was not statistically significant (*p* = 0.13).

### 3.4 Antibiotic and Antiparasitic Use

Goats with a history of antibiotic use had a lower positivity rate (15%) compared to those without (85%), but the association was not statistically significant (*p* = 0.21). However, antiparasitic use showed a significant impact: goats treated under veterinary prescription had a lower positivity rate (32%) than those receiving non‐prescribed treatments (68%) (*p* = 0.02).

### 3.5 Manure Management

Manure disposal practices were significantly associated with *E. coli* prevalence. Goats from farms practicing open disposal had a higher positivity rate (72%) compared to those using composting methods (28%) (*p* = 0.003), suggesting that improved manure management may reduce environmental contamination and bacterial transmission.

### 3.6 Human–Animal Interaction

High‐frequency contact with children and women was associated with increased *E. coli* positivity (66%) compared to low‐contact households (34%) (*p* = 0.04). Shared living spaces and utensils, along with limited use of protective gear, were common in high‐contact settings, potentially contributing to cross‐contamination.

### 3.7 Environmental Exposure and Seasonality

**FIGURE 2:**
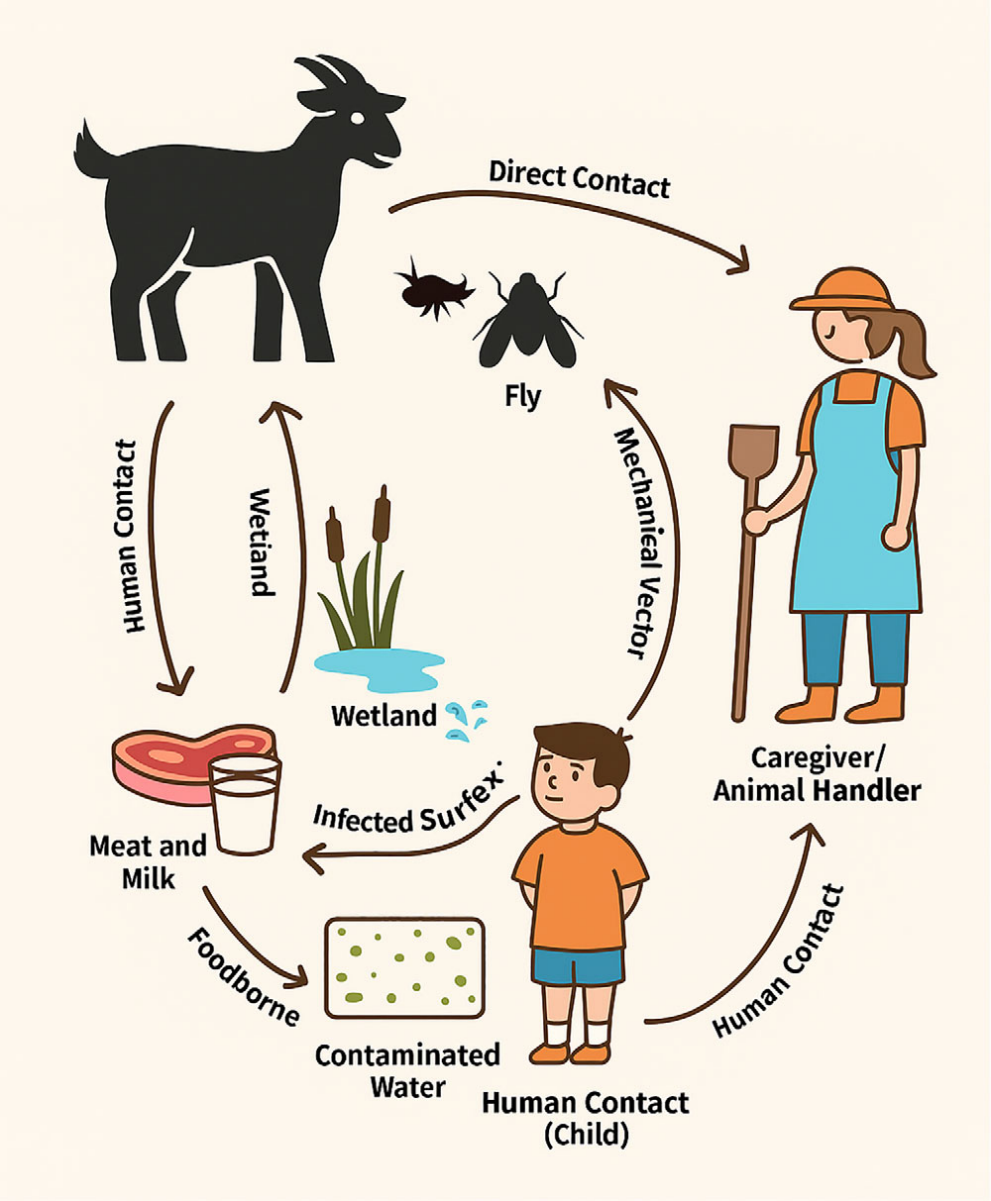
Illustrated (artificial intelligence generated) pathway of zoonotic transmission of *Escherichia coli* from Black Bengal goats to humans. The diagram highlights multiple routes including direct contact with infected animals, mechanical transmission via flies, environmental contamination through wetlands and infected surfaces, and foodborne exposure from contaminated meat and milk. Human contact particularly among children and caregivers is facilitated by shared living spaces, utensils, and lack of protective gear, increasing the risk of infection. Seasonal and environmental factors such as waterlogging and vector density further amplify transmission potential.

Environmental factors played a notable role in *E. coli* prevalence. Goats housed near waterlogged areas or wetlands had a significantly higher positivity rate (71%) than those in dry zones (29%) (*p* = 0.01). Vector density, particularly flies, was higher in wetland‐adjacent farms. Seasonal variation also influenced bacterial shedding: the monsoon season showed significantly higher positivity (69%) compared to the dry season (31%) (*p* = 0.005), likely due to increased moisture and vector activity (Figure 2).

### 3.8 Risk Factor Analysis

Because only one isolate (0.3%) was confirmed as *stx2*‐positive (STEC), statistical analysis of STEC‐specific risk factors was not feasible. Therefore, the associations presented in Table 3 reflect factors associated with overall *E. coli* carriage in goats rather than confirmed STEC infection.

Risk factor analysis for *E. coli* carriage revealed strong associations between *E. coli* positivity and several human–animal interaction parameters (Table 4). Goats from households where children frequently handled animals showed an *E. coli* positivity of 24.1%, compared to 11.4% in households with limited contact (OR = 2.45, 95% CI: 1.08–5.56, *p* = 0.031). Similarly, shared living spaces (e.g., goats housed adjacent to kitchens or bedrooms) were linked to higher *E. coli* positivity rates (21.7%) than those kept in separate sheds (10.5%) (OR = 2.36, *p* = 0.045). Use of protective gear during handling was rare (<10%), and no significant protective effect was observed due to low adoption.

**TABLE 4:** Risk factors associated with *E. coli* carriage in Black Bengal goats.

Risk factor	Category	Prevalence (%)	Odds ratio	*p* value
Housing type	Open vs. semi‐enclosed	26.7 vs. 12.5	2.52	0.022
Child contact frequency	Frequent vs. limited	24.1 vs. 11.4	2.45	0.031
Shared living space	Yes vs. no	21.7 vs. 10.5	2.36	0.045
Proximity to waterlogged areas	Yes vs. no	28.6 vs. 9.8	3.72	0.008
Mixed livestock species	Present vs. absent	23.5 vs. 12.1	2.23	0.041
Vector density (monsoon)	High vs. low	25.0 vs. 13.3	2.17	0.038
Season (July–Sept vs. others)	Monsoon vs. dry/winter	27.8 vs. 10.2	3.12	0.015

Environmental factors also played a critical role. Farms located near waterlogged areas or wetlands had a significantly higher *E. coli* positivity rates (28.6%) compared to elevated dry zones (9.8%) (OR = 3.72, *p* = 0.008). The presence of mixed livestock species (cattle, poultry, ducks) was associated with increased *E. coli* positivity (23.5% vs. 12.1%, OR = 2.23, *p* = 0.041). Seasonal trends showed peak *E. coli* positivity during July–September, coinciding with high humidity (85%–95%) and rainfall (>2500 mm), consistent with the findings from Rumi et al. (2025) and Islam et al. (2008).

## 4 Discussion

The present study aimed to investigate the prevalence of Shiga toxin–producing sorbitol non‐fermenting STEC and general *E. coli* carriage in clinically sick Black Bengal goats. The overall prevalence of goats harbouring sorbitol non‐fermenting *E. coli* was 4.3% based on RAJ swabs cultured on CT‐SMAC. However, the proportion of goats confirmed positive for STEC (carrying the *stx2* gene) was notably low (0.3%). These findings indicate that although Black Bengal goats may serve as reservoirs of sorbitol non‐fermenting *E. coli*, confirmed STEC carriage is relatively uncommon compared with earlier reports in goats and cattle (Gupta 2013; DebRoy and Roberts 2006).

The method used for identifying presumptive STEC had a significant impact on the isolation rate. In this study, colourless colonies on CT‐SMAC were initially considered potential STEC due to their sorbitol non‐fermenting phenotype. However, subsequent PCR analysis demonstrated that most isolates did not harbour *stx1*, *stx2*, or *hlyA/ehxA* genes. This highlights that phenotypic identification alone is insufficient, and the presence of colourless colonies on CT‐SMAC should not be used to infer specific STEC serogroups, such as O157, without molecular confirmation.

Compared with previous studies, the prevalence observed here is lower. Earlier reports have documented STEC prevalence ranging from 1.5% to 4.5% based on virulence gene detection (DebRoy and Roberts 2006), approximately 6% in healthy Black Bengal goats (Gupta 2013), and up to 10% in goats from slaughter markets in Bangladesh (Islam et al. 2008). Such variation may be attributed to differences in sampling strategies, animal health status, geographic location, and diagnostic techniques, including the use of more sensitive methods such as immunomagnetic separation.

The expanded molecular characterization in this study revealed a broader spectrum of virulence markers beyond the classical *stx* genes. The detection of *ehxA* in the *stx2‐*positive isolate is particularly significant, as enterohaemolysin is frequently associated with highly pathogenic STEC strains. Notably, an isolate from an apparently healthy goat carried the *saa* and *subA* genes while lacking *stx* and *eae*. The *saa* gene encodes an autoagglutinating adhesin commonly found in locus of enterocyte effacement (LEE)‐negative STEC, whereas *subA* encodes a potent subtilase cytotoxin. These virulence factors are often present in atypical STEC strains that do not possess the *eae* gene but are still capable of causing severe human disease, including HUS.

The detection of only a single *stx2*‐positive isolate supports earlier observations that ruminant STEC isolates often carry a single Shiga toxin gene rather than multiple toxin variants (Islam et al. 2008). Moreover, the absence of isolates harbouring multiple key virulence genes (e.g., *stx1*, *stx2*, and *ehxA*) indicates that highly virulent STEC combinations may be rare in this population.

Beyond STEC, the overall isolation rate of *E. coli* was higher in clinically affected goats, suggesting a possible association with diarrhoeal disease. However, the absence of *E. coli* in a substantial proportion of samples may be due to prior antimicrobial use, which is common but often undocumented in field conditions.

Several host‐ and management‐related factors were significantly associated with *E. coli* carriage. Adult goats showed higher positivity rates than young goats, likely reflecting increased environmental exposure over time. Diarrhoeic and cachectic animals had significantly higher prevalence, supporting the role of compromised health and immunity in facilitating colonization. Environmental and management factors, including mixed feeding systems, open manure disposal, and housing near wetlands, were also associated with increased bacterial prevalence, likely due to enhanced exposure to contaminated environments.

Human–animal interactions further influenced bacterial carriage, with higher prevalence observed in households with close contact between goats and family members. This finding raises important public health concerns, as zoonotic transmission of pathogenic *E. coli* can occur through direct contact or environmental contamination.

Overall, while the prevalence of STEC in Black Bengal goats was low, the detection of diverse virulence gene profiles, including atypical markers such as *saa* and *subA*, underscores the potential zoonotic significance of these animals. These findings highlight the importance of combining phenotypic and molecular methods for accurate detection and emphasize the need for improved hygiene, management practices, and surveillance strategies.

Future studies should focus on comprehensive molecular characterization, including serotyping and antimicrobial resistance profiling, to better understand the epidemiology, pathogenic potential, and public health implications of *E. coli* strains circulating in goat populations.

## 5 Conclusion

Based on phenotypic characterization of RAJ samples on CT‐SMAC, 4.3% of Black Bengal goats were identified as carriers of sorbitol non‐fermenting *E. coli*. By employing a more comprehensive molecular approach, this study identified not only *stx2* but also accessory virulence markers, including *ehxA*, *saa*, and *subA*. These findings demonstrate that Black Bengal goats in Bangladesh harbour diverse STEC‐related virulence factors, including those associated with severe disease in LEE‐negative strains. The isolation rate of non‐STEC *E. coli* was significantly higher in clinically sick (diarrhoeic) goats compared with apparently healthy animals. Although the overall STEC carriage rate is low, the presence of these virulence markers highlights the zoonotic potential of these animals.

Furthermore, the identified host, environmental, and management‐related risk factors associated with *E. coli* carriage emphasize the importance of improved hygiene and farm management practices in smallholder systems. Overall, these results underscore the need for incorporating a broader panel of virulence genes in future surveillance programs to better assess public health risks and understand the epidemiology of STEC in goat populations.

## Author Contributions

**Shubho Sutradhar**: conceptualization, methodology, investigation, formal analysis, data curation, writing – original draft preparation. **Shormin Akter**: methodology, data curation, writing – review and editing. **Sayeda Ayesha Sultana**: investigation, data curation. **Sudeb Sarker**: investigation, methodology, data curation. **Md. Rigan Molla**: supervision, software, resources. **Moshammat Naifa Begum**: validation, visualization. **Marina Ghosh**: investigation, resources. **Sharifuzzaman**: supervision, conceptualization, study design, writing – review and editing.

## Funding

The authors have nothing to report.

## Ethics Statement

All experimental procedures adhered to institutional guidelines for the ethical care and use of laboratory animals. The study protocol was reviewed and approved by the Ethics Committee of Chattogram Veterinary and Animal Sciences University (Approval No. CVASU‐EC/2023).

## Conflicts of Interest

The authors declare no conflicts of interest.

## Data Availability

Data are available from the corresponding author on request.
